# Mexican Psychiatric Trainees’ Attitudes Towards People with Mental Illness: A Qualitative Study

**DOI:** 10.1007/s10597-021-00907-5

**Published:** 2021-10-30

**Authors:** Emmeline Lagunes-Cordoba, Roberto Lagunes-Cordoba, Ana Fresan-Orellana, Jorge Gonzalez-Olvera, Manuela Jarrett, Graham Thornicroft, Claire Henderson

**Affiliations:** 1 Health Service and Population Research Department, King’s College London, Institute of Psychiatry, Psychology and Neuroscience, De Crespigny Park, London, SE5 8AF UK; 2 Instituto de Investigaciones Psicológicas, Universidad Veracruzana, Xalapa, Veracruz Mexico; 3 Instituto Nacional de Psiquiatría “Ramón de la Fuente Muñíz”, Mexico City, Mexico; 4 Comisión Nacional Para la Prevención de Adicciones, Mexico City, Mexico; 5 School of Health Sciences, City, University of London, London, UK; 6 Centre for Global Mental Health and Centre for Implementation Science, King’s College London, Institute of Psychiatry, Psychology and Neuroscience, London, UK

**Keywords:** Stigma, Attitudes, Psychiatrists, Trainees

## Abstract

Despite their training, psychiatrists have been found to have negative attitudes towards people with mental illness, including the patients they treat. Similarly, studies focused on service users have identified psychiatrists as a source of stigma. Even though negative attitudes in psychiatrists have been identified in different countries and settings, in Mexico the attitudes of these professionals have never been assessed. Because of this, we invited psychiatric trainees from a hospital in Mexico to participate in individual interviews to describe their opinions regarding mental health-related stigma, to evaluate their attitudes towards people with mental illness and to identify factors that could be influencing their attitudes. Interviews were audio recorded, transcribed and analysed using thematic analysis. A total of 29 trainees participated in the study. The results suggested that trainees recognised psychiatrists can have negative attitudes towards people with mental illness, such as poor empathy, judgement and labelling, and mainly towards patients considered difficult and with borderline personality disorder. Participants recognised these attitudes can influence their relationship with patients, and considered it is necessary to develop interventions to improve their own attitudes and reduce mental health stigma. From this study we concluded Mexican psychiatrists are not free from stigma towards people with mental illness. However, Mexican psychiatric trainees are interested in improving their attitudes and reactions towards their patients.

## Introduction

Despite their specialised knowledge and training, psychiatrists have been recognised as holding stigmatising attitudes and behaviours, as well as stereotypes and desire for social distance, from people with mental health problems (Gras et al., 2014; Lauber et al., 2006; Loch et al., 2013; Nordt et al., 2006). Some studies have even shown that mental health professionals have more pessimistic views about recovery than members of the general population (Hugo, 2001; Jorm et al., 1999; Schulze, 2007; Thornicroft et al., 2010). This might be explained by a phenomenon known as “physician’s bias”, which refers to the bias caused by the continuous contact physicians have with patients who do not fully recover, along with the lack of contact with fully recovered patients. (Thornicroft et al., 2007; Thornicroft, Rose & Mehta, 2010). Similarly, service users have reported feeling stigmatised by mental health professionals, with a perceived lack of interest in their personal history; diagnoses given with a negative prognosis and without empathy (Horsfall et al., 2010; Schulze, 2007; Schulze & Angermeyer, 2003); receiving limited information; being treated “like children”; and being excluded from decision making (Schulze, 2007; Thornicroft et al., 2007).

Although stigma research has proliferated over the last two decades, studies conducted in low and middle-income countries are relatively scarce (Semrau et al., 2015; Thornicroft et al., 2016, 2019), and there is still little on mental health professionals (Heim et al., 2019). In Mexico, a middle-income country (Wang et al., 2007), most stigma research has focused on public stigma (Mora-Rios et al., 2013a, b; Robles-Garcia et al., 2013). However, three studies on medical and psychology students (Fresan et al., 2012; Fresán et al., 2013; Vargas-Huicochea et al., 2017) reported that these groups considered people with schizophrenia and bipolar disorder to be aggressive and dangerous. Similarly, Mora-Rios & Bautista (2014) reported that service users in Mexico considered health care personnel as the second source of stigma and discrimination, preceded only by their family members. Similarly, in a recent study focused on mental health service users, participants considered psychiatrists can have negative attitudes towards people with mental illness, which could in consequence impact on their treatment (Lagunes-Cordoba et al., 2020). This suggests mental health professionals in Mexico, including psychiatrists, might have negative attitudes towards people with mental illness, including the patients they treat, attitudes which could potentially impact on the care they provide to their patients. However, to our knowledge, there are no studies focused on Mexican psychiatrists’ attitudes,

To address this gap, we developed this study, of which main aim was to explore the attitudes of Mexican psychiatric trainees towards people with mental illness; to describe their opinions regarding mental health-related stigma; and to identify potential factors that could influence their attitudes. We also aimed to use the results of this study to develop a targeted anti-stigma intervention for this population (Lagunes-Cordoba et al., 2021).

## Methods

### Design and Setting

This was a qualitative study for which we invited psychiatric trainees from a psychiatric hospital in Mexico City. This hospital accepts each year an average of 20 doctors, who will undertake a 4 years’ training programme to become general adult psychiatrists. Additionally, this hospital annually admits one to three psychiatric trainees to each of its 13 subspecialty programs (e.g. old age psychiatry, eating disorders, or child and adolescent psychiatry). At the time of the study, there were 114 trainees registered within the hospital. The study was approved by the Psychiatry, Midwifery and Nursing Research Ethics Committee of King´s College London (HR­15/16­2763) and by the Research Ethics Committee of the hospital where this research was conducted.

### Participants

All participants were psychiatric trainees registered at the hospital where the study took place, and who agreed to participate. We included trainees of all academic years and rotating in the different clinics, as we used theoretical sampling, which facilitates identifying diverse possibilities that are theoretically relevant to our research questions. We also used theoretical sampling because attitudes of trainees might change over the course of their training and be influenced by experiences with different services or patient groups. This sample strategy also allowed for theoretical saturation, as participant’s recruitment ended when we were no longer obtaining new information from individual interviews (Guest et al., 2006).

### Recruitment Data and Collection

All registered trainees were invited to participate to the study through circular emails which included the information sheet, a copy of the consent form and information about how to contact the principal researcher (ELC) to express their interest and arrange an individual interview. After the purpose of the study was explained, interested trainees were asked to sign the consent form and were reminded that they could drop the study or withdraw their information at any time, but no longer than three months after they participated in the study. One trainee who booked an interview later declined his participation, but no participant withdrew their consent after interviews were conducted.

All interviews were conducted by the principal researcher, within the premises of the hospital using open-ended questions from a topic guide. The topic guide included questions regarding general knowledge of mental health stigma, stigma in mental health professionals, witnessing stigmatising attitudes and behaviours in fellow colleagues, and possible solutions to reduce stigmatising attitudes and discrimination. A copy of the topic guide is available upon request. Interviews were audio recorded and transcribed verbatim for its later analysis.

### Data Analysis

Interviews were transcribed and analysed in Spanish using thematic analysis with both, inductive and deductive approach at a semantic level, as the coding process initially involved identifying codes aimed to answer our research questions, but an inductive process was later followed to establish new categories and themes (Braun & Clarke, 2006). Data analysis was first conducted by the principal researcher and later reviewed by another member of the research team (MJ) to establish credibility and compare identified codes, themes and sub-themes, until agreement was reached. A total of 28 codes were grouped into categories; these were arranged into subthemes, which were then organised into three overarching themes. Categories, subthemes and quotes used to exemplify these themes were translated to English. See Table 1 for examples of trigger questions, and the resulting categories, subthemes and themes.

**Table 1:** Categories, Subthemes and themes emerged from trigger questions

Example of trigger questions	Categories	Subthemes	Themes
Have you ever witnessed any colleague treating patients in a disrespectful way or a manner you consider unprofessional?	Poor empathy	Stigma within psychiatrists relationship with patients	Psychiatrists as a source of stigma
	Prejudice		
	Paternalism		
Do you think psychiatrists are free from stigma towards the patients they treat?	Labelling	Stigma outside direct contact with patients	
	Prejudice		
	Avoidance		
Do you think there are patients psychiatrists are more likely to treat differently?	Poor empathy	Stigma towards people with borderline personality disorder	
	Dismissal		
	Rejection		
How do you think psychiatrists react mental towards colleagues with mental health problems?	Rejection	Stigma toward psychiatrists with mental disorders	
	Labelling		
	Concealment		
Why do you think your colleagues react/behave in that way? Have you ever receive any class or any training about this subject? Have you ever felt pressured to act or behave in a certain way related because of other colleagues?	Burnout	Factors related to psychiatrists	Causes of stigma in psychiatrists
	Frustration		
	Prior experiences		
	Denial		
	Ignorance		
	Difficult patients	Factors related to patients	
	Borderline personality		
	Work overload	Institutional factors	
	Lack of training/ supervision		
	Normalisation		
	Negative influence of colleagues/ peer pressure		
Do you think stigma in psychiatrists is something that should be addressed? How?	Training from year 1	Proposed interventions to improved attitudes in psychiatrists	Interventions to improve psychiatrists’ attitudes
	Mandatory training		
	Participation of patients		
	Psychotherapy		
Do you think there are any barriers to deliver an anti-stigma intervention for this population?	Time and work overload	Barriers for implementing an anti-stigma intervention	
	Conflict between academic and clinical work		
	Lack of interest		
Do you think psychiatric trainees will be willing to participate in an anti-stigma intervention?	Patient benefits	Potential outcomes	
	Personal benefits		

## Results

A total of 29 trainees (25.4%) participated in the study, 16 men and 13 women. There were four trainees from year one, nine from year two, ten from year three, four from year four and two from a high speciality program. Interviews lasted an average of 15 min (range 9–30 min). Saturation was considered reached around the 20th interview, but we included more participants as by that point most participants were males and from the second and third academic years. There was no debrief with participants, but the information sheet included a statement saying that if they would like to know the results of the study, they could contact the principal researcher. We identified ten subthemes from 28 categories, which were grouped into three overarching themes: psychiatrists as a source of stigma, causes of stigma in psychiatrists and interventions to improve attitudes in psychiatrists.

### Psychiatrists as a Source of Stigma

Discussions about stigma in psychiatrists led to four main subthemes: stigma within psychiatrists’ relationship with patients, stigma outside direct contact with patients, stigma towards people with borderline personality disorder, and stigma towards colleagues with mental disorders.

#### Stigma within Psychiatrists´ Relationship with Patients

##### Poor Empathy and Judgement

Participants described colleagues with no patience or interest in hearing what patients have to say, as they are often rushed or interrupted if they don’t talk about what psychiatrists want to hear. Judgments of patients´ personal lives were also commonly mentioned; one participant observed a colleague talking in a derogatory manner in front of patients, arguing that their psychosis or mania meant that they would not remember this.

*We don´t have patience for patients that we know are very neurotic, very apprehensive or with a personality disorder; we try to move to other issues when patients are expressing what is important for them, we skip that because we don´t care about that, they are not telling our criteria* (Male, 27 years).

##### Prejudice and Paternalism

Participants also consider that a common practice is the lack of inclusion of patients in important decisions and the lack of explanations about patients´ treatment, even when this includes physical restriction. Some considered this could be consequence of paternalism or even personal prejudice.

*…It is like, you are going to take this, and that´s it. I think that not including them in their treatment is maybe not direct stigma, but we are saying that we need to decide for them because they don´t have the capacity or they don´t know which medication is the best* (Female, 28 years).

Discouragement of patients to pursue certain careers because their diagnoses and treating patients as if they were children were also reported by a couple of participants as examples of stigma.

#### Stigma by Psychiatrists Outside Direct Contact with Patients

##### Labelling

Most participants stated that they perceived more negative attitudes in psychiatrists when they were not in front of a patient but with other colleagues, and the most common reactions observed in other psychiatrists that participants reported were labelling and calling patients by their diagnoses in a derogatory manner.

*Disorders like borderline, we call them border patients. I mean, we depersonalise the individual, we call them by their diagnosis not by their name* (Male, 30 years).

Some reported discussions between colleagues extended to devaluation, mockery and use of offensive nicknames to refer to their patients. However, one participant considered that this was limited to private conversations, so did not necessarily affect patients.

##### Prejudice and Avoidance

There were various accounts of psychiatrists referring to patients in a negative way or expecting them to behave in a certain way, even before meeting patients because of their diagnosis. This prejudice can lead psychiatrists to dismiss patients´ symptoms and to even avoid treating patients with diagnoses they consider difficult.

*this woman arrived and nobody wanted to see her, there were comments in the emergency room: what a pain, this is tiring, this is boring, this one wants to kill herself again; I mean, we dismissed patient´s symptoms* (Male, 30 years).

#### Stigma Towards People with Borderline Personality Disorder

Participants considered that most negative reactions they have were related to patients with borderline personality disorder (BPD). According to participants, patients with this diagnosis are often considered as difficult and demanding, and their symptoms are often dismissed or faced with little empathy.

*When they come with suicidal thoughts, we make them wait longer. We say: ‘surely while she waits the acting out would have passed’. I have observed that lots.* (Female, 29 years).

A couple of participants reported that these negative attitudes towards people with BPD are not limited to patients, as they have observed other psychiatrists assuming people in the street have a BPD because of the way they look, or devaluating fellow colleagues because they think a colleague could have this diagnosis.

#### Lack of Empathy Towards Other Psychiatrists with Mental Disorders

##### Labelling and rejection

Some participants (around 50%) reported witnessing other psychiatrists undermining or using certain diagnoses to describe colleagues if they know or suspect the individual had a mental disorder.

*I think we don´t tolerate that (cases of mental illness in other psychiatrists) very well, and even if they are understood, at the same time we push them away, and we attribute their actions to their mental illness: this one got depressed or this one is a border* (Female, 27 years).

##### Concealment

Some participants considered that they feel that they are “not allowed” to have a mental disorder or that they need to hide this to avoid criticism or rejections form their colleagues and even from the authorities.

*Some of my colleagues are already taking antidepressants, but they keep it as a secret because they don´t want to be regarded as weak or vulnerable* (Female, 33 years).

### Causes of Stigma in Psychiatrists

Causes of stigma were classified into three subthemes: factors associated with psychiatrists, factors associated with patients and institutional factors.

#### Factors Associated with Psychiatrists

##### Burnout and Frustration

Most participants considered that fatigue and burnout were the main reasons why psychiatrists have poor empathy and negative attitudes towards their patients. Similarly, frustration about not getting expected clinical results, or improvements in their patients, was often mentioned as possible causes.

*I think it could be because we are saturated, our time is saturated, they book us patient after patient* (Female, 30 years).

*Maybe … because we have very difficult patients that don´t accept treatment, maybe we get frustrated because we don´t get our therapeutic objectives* (Female, 28 years).

##### Previous Experiences

Some trainees considered that negative experiences with people with mental illness, medical training, and cultural background could be related to prejudice and rejection.

*Because we start our training in mental health when we already have an opinion. I think we don´t start in blank... we are already influenced by the media* (Female, 28 years).

##### Ignorance and Lack of Awareness

Most trainees mentioned they had received little or no training related to stigma, and some considered that this along with ignorance, was related to negative attitudes towards their patients. Some trainees also reported that sometimes they did not feel adequately prepared to deal with some types of patients, notably those with BPD.

*I don´t think everything is due to lack of empathy, I think there is also a lack of information… which kind of behaviours we have toward our patients and how they can be affected by it* (Female, 29 year).

Other factors mentioned by participants were denial, negative counter-transference, desensitisation and even narcissistic or other personality traits in psychiatrists.

#### Factors related to Psychiatric Patients

##### Difficult Patients

Many trainees considered that negative attitudes are often caused by dealing with people they regarded as difficult patients. According to participants dealing with a “difficult patient” is not only more tiring and frustrating; it could also lead to rejection of other patients with the same diagnosis.

*I think this happens because there might be things related to their illness that make them more difficult to treat than others, … then you start having this idea, that they will be more difficult or more tiring, or that they could be aggressive* (Male, 29 years).

##### Borderline Personality Disorder

Although having BPD was not considered itself as a cause of stigma in psychiatrists, there were many reports suggesting that most negative attitudes in psychiatrists are related to patients with this diagnosis. Therefore, treating someone with diagnosis of BPD was recognised as a factor for negative attitudes in psychiatrists.

*When a patient with BPD comes and says that she wants to be hospitalised, they (psychiatrists) say, she has nothing… they say, she just had an argument with her boyfriend, like implying she is manipulating the staff, implying she doesn’t have an illness or that she is doing this to annoy us* (Male, 29 years).

Other characteristics of what trainees considered as “difficult patients” were violent or aggressive behaviour towards psychiatric staff and low treatment adherence.

#### Institutional Factors

Most trainees attributed some of their negative attitudes to factors related to the hospital or their training: work overload, lack of training and supervision, negative influence of colleagues, and peer pressure.

##### Work Overload

Work overload was considered a major cause of tiredness and burnout by many participants. There was a shared feeling that authorities are more focussed on increasing the number of patients than improving the quality of care they received.

*There are some parts in our residency when as a result of seeing so many patients, we obviously get tired and this same resentment and tiredness can make you try to get even with a patient or to think badly about them, with prejudice* (Male, 29 years).

##### Lack of Training and Supervision

Some participants considered that their host institution failed to provide proper supervision or specific training. Some considered that the suspension of Balint groups, at which trainees were able to discuss difficult cases with colleagues, had worsened their attitudes towards their patients.

*How is it possible that they are teaching me everything about psychiatry, but they don´t teach me how to not treat a patient badly?* (Male, 27 years).

##### Normalisation

Even though some participants stated that they are aware that some of their common reactions could be regarded as stigmatising, they considered that normalisation plays an important role in perpetuating these negative attitudes and reaction towards people with mental illness. Most trainees reported that complaining about patients or calling them by names or diagnoses was tolerated as normal practice.

*At the beginning when this used to happen (mocking of patients), I used to feel that it wasn´t fine, but oddly enough, it´s something that grabs you, it is something that becomes usual, it gets normalised* (Male, 28 years).

##### Negative Influence of Colleagues and Peer Pressure (Hidden Curricula)

Stigmatising attitudes and behaviours of other psychiatrists were considered causal and contributing factors. As trainees not only felt pressured to imitate others, but they also ended up endorsing such attitudes and repeating what they learned from colleagues.

*When they say that a patient tends to lie or uses doctors or manipulates them, then I feel I have to follow that line of treatment, and not be able to treat the patient the way I would like”* (Female, 26 years).

### Interventions to Improve Psychiatrists’ Attitudes

These were grouped in three subthemes: proposed interventions to improve attitudes in psychiatrists, barriers for implementing an anti-stigma intervention and potential outcomes.

#### Proposed Interventions

Most participants suggested the inclusion of early and mandatory training related to mental health stigma in their curricula. Some participants also suggested that courses should be taught each year to reinforce learning.

*I think we shouldn´t be given an option, it’s something that should be part of our training, because, … It’s not if you want or not treat patients badly or having stigma towards our patients* (Male, 27 years).

Suggestions included involving patients, or their personal testimonies, to increase trainees’ awareness of their attitudes. Trainees also suggested receiving training focused on the management of patients with personality disorder or on controlling counter-transference feelings and one´s own stigmatising attitudes. Finally, some considered psychiatrists should receive psychotherapy as attitudes could be related to their own personality traits and, because there is also a need to take care of psychiatrists´ own mental health.

*I think we need therapy, … because we are exposed to difficult things, and I think this would help us to identify attitudes like, I don´t like this person, and avoid generalising* (Male, 27 years).

#### Barriers for Implementing an Anti-stigma Intervention

Time or the lack of it was considered the main barrier to implement an anti-stigma intervention in this particular setting. Trainees considered that their work overload does not leave them with spare time to take part in any intervention, not even Balint groups. Conflict between academic and clinical work was also considered an important barrier not only for implementing an anti-stigma intervention, but also for taking part in their academic activities.

*The problem is the education department wants something, but we are told to do more work/clinical activities, more time is demanded from us, and they (admin department) limit our academic part* (Male, 27 years).

A lack of awareness and interest in the subject, were also considered as possible barriers, although these were also mentioned as causes of stigma in psychiatrists.

*Maybe people don´t want to officially recognise that there is stigma from psychiatrists themselves, therefore they don´t want to open a space* (Male, 29 years).

#### Possible Outcomes

Participants considered that patients would benefit from an anti-stigma intervention for psychiatrists because reducing stigma could improve communication and therapeutic relationships, which in consequence could lead to improvements in care and life quality. Participants also considered that an anti-stigma intervention could equip psychiatrists to help patients deal with other sources of stigma such as family members and employers. Similarly, trainees considered that an intervention could positively impact their future practice, resulting in fewer complaints and even better job opportunities.

*Reducing our stigma would help us to understand them better and to provide them with a better treatment, so at the end of the day, they could have a better life* (Male, 26 years).

*Our clinical practice would be more ethical and the patient would be benefited, I think even us as psychiatrists, we would be more satisfied with our jobs* (Female, 33 years).

## Discussion

Results from this study suggested that Mexican psychiatric trainees not only hold some negative attitudes towards people with mental illness, but they also recognise the presence of such attitudes in themselves and their colleagues, and the need to address them to improve patient care.

### Negative Attitudes Towards People with Mental Illness

Our results suggested that the main negative attitudes of psychiatric trainees in Mexico are poor empathy, prejudice, paternalism, avoidance and labelling; attitudes participants recognised in themselves and/or their colleagues. Trainees considered psychiatrists are often not interested in the personal life of their patients and considered they often judge and question patients´ decisions or lifestyles. This lack of interest in mental health professionals has been described by service users in other studies (Buizza et al., 2007; Hamilton, 2016; Schulze & Angermeyer, 2003); however, direct judgement and criticism have been attributed more often to other health care staff rather than to mental health professionals (Schulze & Angermeyer, 2003).

Paternalism has also been identified in other studies in which service users reported not receiving enough information about their diagnosis or not being included in important decisions (Hamilton, 2016; Buizza et al., 2007; Schulze & Angermeyer, 2003). This paternalistic attitudes among professionals may reflect a belief that patients are incapable of taking their own decisions. Other studies that have assessed mental health professionals´ attitudes (Hugo, 2001; Lauber et al., 2004, 2006; Yuan et al., 2017) have also found that these professionals can have the same negative stereotypes and desire for social distance as does the general population. Some authors have argued that considering people with mental illness as dangerous, socially disturbed and not highly skilled (Lauber et al., 2006; Wahl & Aroesty-Cohen, 2010) could cause psychiatrists to avoid or regard their patients as not worthy of receiving care.

Overall, our results suggested that trainees had more negative attitudes towards patients they consider difficult, aggressive or with low treatment adherence. However, most trainees reported more negative attitudes towards people with borderline personality disorder (BPD). Other studies have also reported that psychiatrists have poor empathy towards people with BPD as they tend to consider people with BPD as difficult, demanding and with poor prognosis (Black et al., 2011; Bodner et al., 2015; Chartonas et al., 2017; Knaak et al., 2015). Authors of most studies focused on exploring the attitudes of psychiatrists towards patients with BPD have suggested the need to improve training or implement workshops for psychiatrist to change the attitudes of these clinicians, training that might also be necessary to implement for Mexican psychiatrists.

Our results also suggested that psychiatrists have poor empathy towards colleagues known to have mental disorders as they tend to label and reject them. This has led trainees to conceal disorders from their peers, a finding reported in other studies which have identified fear of stigma and of jeopardising their careers as the main causes for concealing a mental disorder in these professionals (Hassan et al., 2013; Knaak et al., 2017; Moutier et al., 2009).

### Factors Associated to Negative Attitudes in Psychiatrists

In keeping with what was reported by our participants, burnout has been considered by other authors as an important factor related to negative attitudes in these professionals (Bayar et al., 2009; Gras et al., 2014; Henderson et al., 2014), as cynicism, an element of burnout, could influence peoples´ beliefs and attitudes. Bayar et al (2009) have also suggested that burnout might be influenced by “physician bias”, which has been identified a factor influencing psychiatrists’ attitudes towards people with mental illness (Thornicroft et al., 2007, 2010).

Paying attention to their own attitudes has been considered an important element for improving mental health professionals´ attitudes towards their patients, because it targets the assumption of mental health professionals as exempt from stigma (Knaak et al., 2017; Lauber et al., 2004; Wahl & Aroesty-Cohen, 2010). This is in line with participants in this study, who reflected that lack of awareness and even denial of their own negative attitudes could be contributing to the persistence and normalisation of stigmatising attitudes.

However, many argued that some of their negative attitudes could be consequences of their patients’ attitudes or diagnoses, in other words, responses to their patients’ behaviours. This coincides with what some authors have suggested, that negative attitudes in psychiatrists, mainly towards people with BPD, could be related to behaviours that can provoke negative but “human” reactions in clinicians (Bodner et al., 2015; Chartonas et al., 2017). However, professionalism includes learning not to react in a ‘normal human’ way if this way is negative, particularly if such reactions contribute to problems such as low adherence to treatment or aggressive behaviour.

Even though some trainees reported negative practices like name-calling were wrong, eventually many adopted this kind of behaviour. This influence could be seen as a hidden curriculum, which refers to those messages implicit through common vocabulary, repeated practices and habits that impact the attitudes and practices of those “learning” a new job (Liao et al., 2014). Hidden curricula seem to be related to the tolerance and acquisition of practices initially considered to be wrong in order to be part of the team (Liao et al., 2014; Martinez et al., 2017). Similarly, trainees’ reports also reflect the use of ‘gallows humour’, a common practice amongst physicians to reduce anxiety and stress (Bennett, 2003). However, this anxiety and stress may be related to lack of skills to manage certain patients, as lack of skills and training may cause professionals to avoid patients or respond inappropriately to them (Bodner et al., 2015; Knaak et al., 2017), with consequent unsatisfactory outcomes and reinforcement of negative attitudes.

Most trainees considered that education on stigma should be part of their training and stated they were willing to participate in an intervention to reduce stigma. However, many considered that other trainees might not be interested, or consider this needed. This contradictory finding could be a consequence of social desirability and/or selection bias in this sample.

### Strengths and Limitations

To our knowledge this is the first study focusing on assessing the attitudes of psychiatric trainees in Latin America towards people with mental illness and exploring their points of view regarding mental health-related stigma in their work settings.

There are some limitations to this study. First, selection and social desirability biases might influence the results; trainees who participated might have been more aware of their own attitudes and therefore more likely to provide “socially desirable” answers. However, having asked trainees to discuss the attitudes and behaviours of their colleagues rather than their own, sought to diminish the effects of social desirability bias. Second, because this study was conducted within the hospital premises, trainees might have been concerned about the anonymity of their participation and potential repercussions of discussing negative reactions of other psychiatrists towards patients. Having said this, participants might had been more prone to responded sincerely as the main researcher was not linked to the hospital, and had no influence on their evaluation or future practice. Finally, as this was a qualitative study conducted in only one hospital, is not possible to generalise results to all Mexican psychiatric trainees. However, residents from the same speciality share the same education programs across Mexico, so there is no reason to expect the findings would differ elsewhere in the country.

Despite its limitations, there are some strengths to our study. First, to our knowledge this is the first study assessing the attitudes Mexican psychiatric trainees have towards people with mental illness and their points of view regarding mental health-related stigma in mental health care settings. Second, our study also identified causes associated to negative attitudes in psychiatrist, and potential barriers for implementing an anti-stigma intervention in a particular setting, which we considered were important elements to later develop a targeted anti-stigma intervention.

### Implications for Research and Practice

Our data suggest that an anti-stigma intervention for psychiatric trainees in Mexico is not only necessary, but also potentially welcomed by this group of professionals. This type of intervention should focus on improving knowledge regarding mental health-related stigma and increasing awareness of its impact on people’s mental health; it should also focus on reducing negative attitudes such as prejudice, labelling, rejection, stereotyping, and normalisation of negative behaviours amongst these professionals. Further organisational and professional culture changes are also needed to reduce stigma and concealment of psychiatrists’ own mental health problems. This includes training focused on handling patients perceived as ‘difficult’ (Corrigan et al., 2012; Thornicroft et al., 2016) and implementing a more reflective learning approach, as its adoption in health care education may lead to better medical practice (Bekas, 2013; Fragkos, 2016; Gibbs et al., 2005). Likewise, reinstalling Balint groups may be a useful tool to reduce stress in physicians (Kjeldman & Holmstrom, 2008), which consequently can lead to improving attitudes and the care they provide to their patients.
